# Supplementary material for: Genomic and In Vitro Phenotypic Comparisons of Epidemic and Non-Epidemic Getah Virus Strains
Source: Viruses. 2022 Apr 30;14(5):942. doi: 10.3390/v14050942 (PMC9145621; doi:10.3390/v14050942)
Supplement: Supplementary file 1 [file viruses-14-00942-s001.zip › viruses-1637938-supplementary.pdf]

viruses

Supplementary Materials

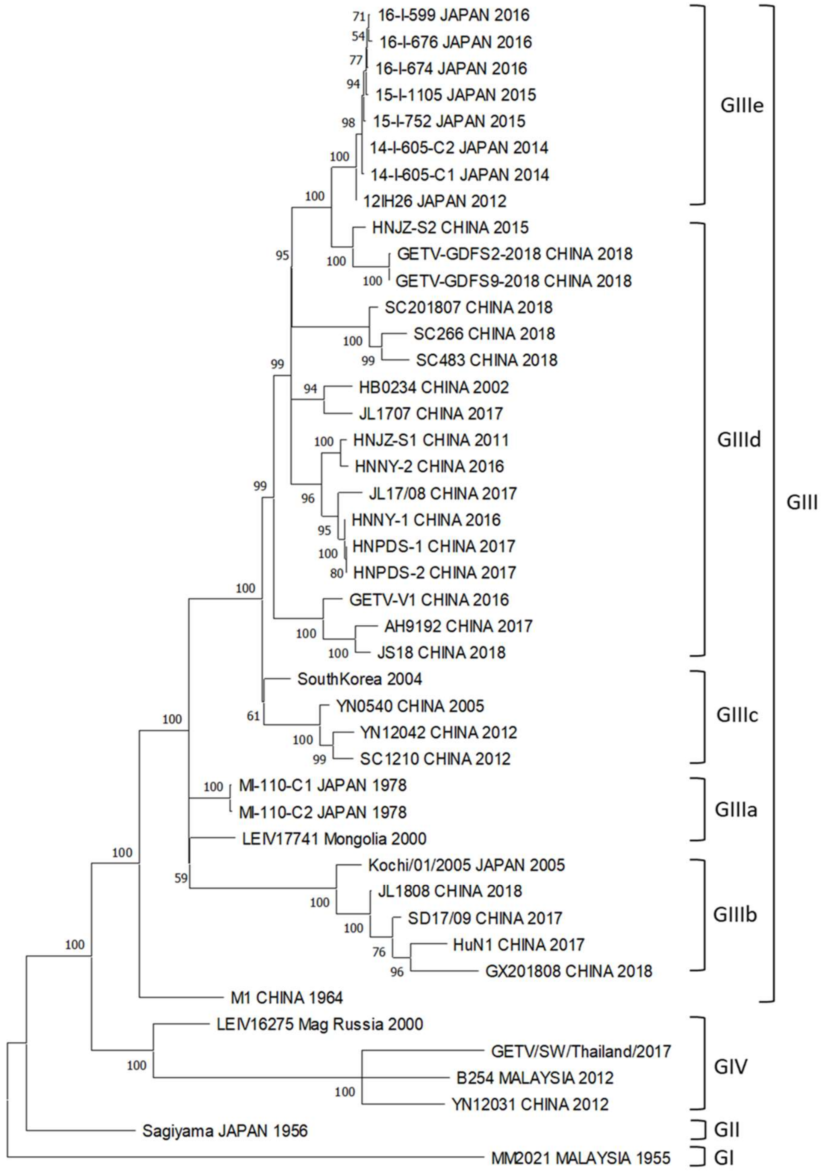

Figure S1. Maximum likelihood phylogenetic analysis of GETV based on the complete coding sequences. The phylogenetic tree was constructed using Mega 10.2.1 and inferred using general time reversible + gamma distributed with invariant sites models (GTR+G+I), based on a bootstrap value of 1000 replicates. The bootstrap support percentage is indicated by the value at each node.

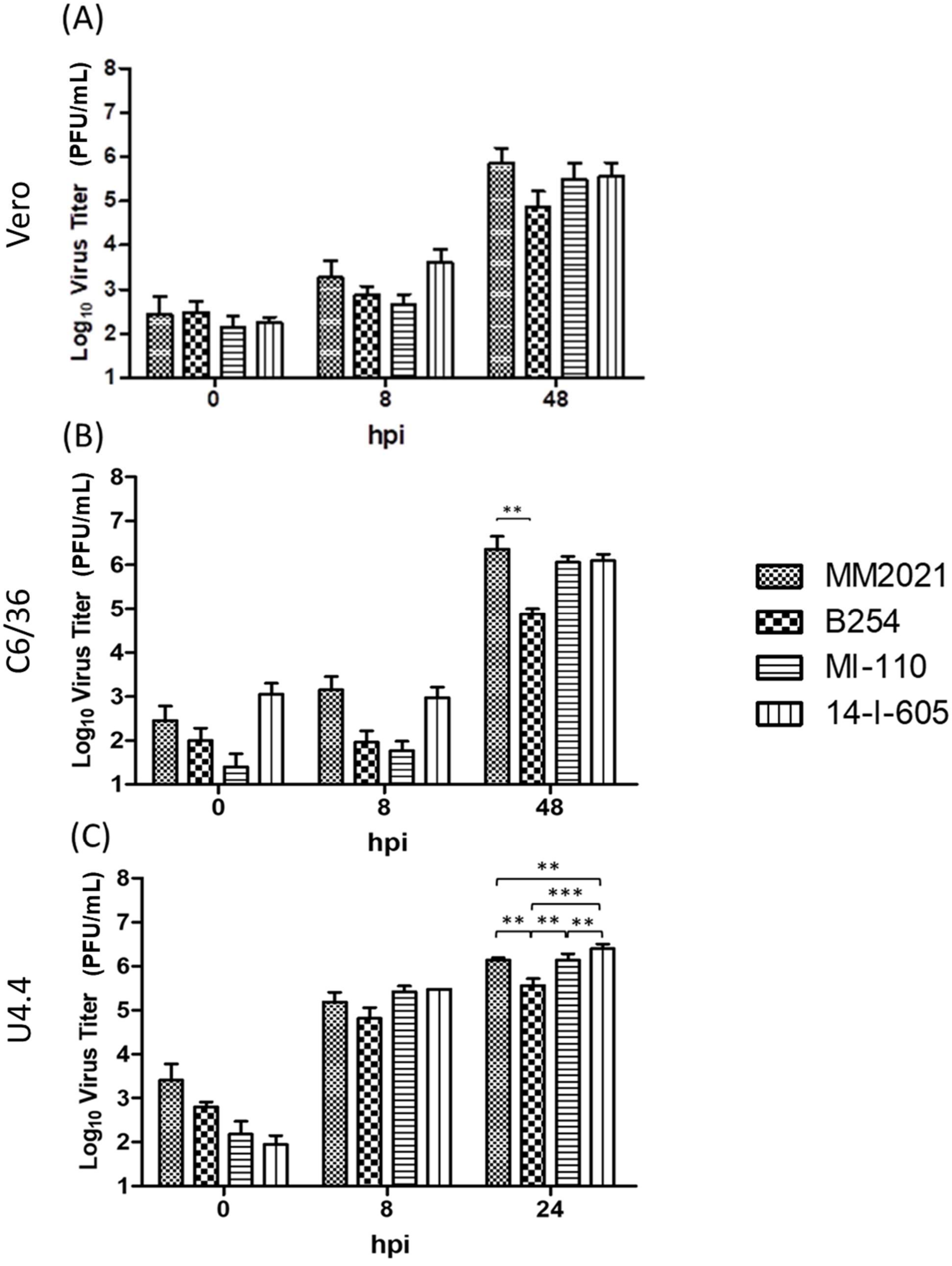
(A)
Vero
Log10 Virus Titer (PFU/mL)
8
7
6
5
4
3
2
1
0
8
48
hpi
(B)
C6/36
Log10 Virus Titer (PFU/mL)
**
0
8
48
hpi
(C)
U4.4
Log10 Virus Titer (PFU/mL)
**
***
** ** **
0
8
24
hpi
MM2021
B254
MI-110
14-I-605

Figure S2. Extracellular infectious viral titers of various GETV strains in the infected (**A**)Vero, (**B**) C6/36, and (**C**) U4.4 cells. In order to validate the infectivity of the extracellular viral samples, plaque assay was performed to measure the infectious viral titers at 0, 8, and 48 hpi. Data plots show the mean infectious viral titers and standard deviation (SD) of three independent replicates. Error bars indicate SD. Asterisks indicate statistical significance (** $p < 0.01$, *** $p < 0.001$) as determined by Bonferroni test.
